# Development of nomogram to predict in-hospital death for patients with intracerebral hemorrhage: A retrospective cohort study

**DOI:** 10.3389/fneur.2022.968623

**Published:** 2022-11-24

**Authors:** Linwang Hu, Jie Yu, Jian Deng, Hong Zhou, Feng Yang, Xiaohang Lu

**Affiliations:** ^1^Department of Neurosurgery, Hunan Provincial People's Hospital, The First Affiliated Hospital of Hunan Normal University, Changsha, China; ^2^Department of Pharmacy, Hunan Provincial People's Hospital, The First Affiliated Hospital of Hunan Normal University, Changsha, China; ^3^Department of Critical Care Medicine, People's Hospital of Ningxia Hui Autonomous Region, Ningxia, China

**Keywords:** systemic immune-inflammation, intracerebral hemorrhage, MIMIC-III database, in-hospital death, nomogram

## Abstract

**Aim:**

This study aimed to investigate the association between systemic immune-inflammation (SII) and the risk of in-hospital death for patients with intracerebral hemorrhage (ICH) in the intensive care units (ICU) and to further develop a prediction model related to SII in predicting the risk of in-hospital death for patients with ICH.

**Methods:**

In this retrospective cohort study, we included 1,176 patients with ICH from the Medical Information Mart for Intensive Care III (MIMIC-III) database. All patients were randomly assigned to the training group for the construction of the nomogram and the testing group for the validation of the nomogram based on a ratio of 8:2. Predictors were screened by the least absolute shrinkage and selection operator (LASSO) regression analysis. A multivariate Cox regression analysis was used to investigate the association between SII and in-hospital death for patients with ICH in the ICU and develop a model for predicting the in-hospital death risk for ICU patients with ICH. The receiver operator characteristic curve was used to assess the predicting performance of the constructed nomogram.

**Results:**

In the training group, 232 patients with ICH died while 708 survived. LASSO regression showed some predictors, including white blood cell count, glucose, blood urea nitrogen, SII, the Glasgow Coma Scale, age, heart rate, mean artery pressure, red blood cell, bicarbonate, red blood cell distribution width, liver cirrhosis, respiratory failure, renal failure, malignant cancer, vasopressor, and mechanical ventilation. A prediction model integrating these predictors was established. The area under the curve (AUC) of the nomogram was 0.810 in the training group and 0.822 in the testing group, indicating that this nomogram might have a good performance.

**Conclusion:**

Systemic immune-inflammation was associated with an increased in-hospital death risk for patients with ICH in the ICU. A nomogram for in-hospital death risk for patients with ICH in the ICU was developed and validated.

## Introduction

Intracerebral hemorrhage (ICH) is the most severe form of stroke characterized by bleeding into the brain parenchyma, which has a 1-month mortality rate of ~50% and has become one of the leading causes of disability and death worldwide (1, 2). A study from the Big Data Observatory platform for stroke in China reported that the in-hospital death rate of ICH was ~5.1%, and the 12-month disability rate was ~29.2% (3). The burden of disease for patients with ICH is huge, especially for those in intensive care units (ICUs). Therefore, an early prediction of death risk in patients with ICH is critical for assessing the severity of the disease and making appropriate treatment decisions.

Perihematomal edema (PHE) could occur within hours after ICH, which may trigger an inflammatory response and lead to a secondary brain injury (4, 5). Previously published studies have shown that inflammatory response played an important role in the development of ICH (6, 7). ICH drives inflammatory responses of microglia and T-cell-mediated in the brain, and these inflammatory responses were related to cytokine release and perihematomal edema, which might cause a poor prognosis after ICH (8). Nowadays, some inflammation parameters have been reported that might be associated with the prognosis of ICH, such as the neutrophil to lymphocyte ratio (NLR), the platelet to lymphocyte ratio (PLR), and the lymphocyte to monocyte ratio (LMR) (9–11). Recently, the systemic immune-inflammation (SII) index, as an integrated indicator based on peripheral lymphocytes, neutrophils, and platelets counts, was considered a new inflammatory marker and could reflect the local immune response and systemic inflammation (12, 13), which has a good prognostic value in a variety of tumors. Huang et al. highlighted that SII was related to the prognosis of patients with cervical cancer, and the nomogram based on SII showed a good predictive value for predicting postoperative survival of patients with cervical cancer (14). Nevertheless, as far as we know, there were few studies to evaluate the relationship between the SII and in-hospital death of patients with ICH in the ICU, and the predictive value of SII on in-hospital death.

Herein, the present study aimed to investigate the association between SII and the risk of in-hospital death for patients with ICH in the ICU and to further combine other prognostic indicators to develop a prediction model in predicting the risk of in-hospital death for patients with ICH.

## Methods

### Data sources and study population

In this retrospective cohort study, all data were derived from the Medical Information Mart for Intensive Care (MIMIC-III) database. MIMIC-III is a large, freely-available database, containing the de-identified health-related data of patients admitted to the ICU of the Beth Israel Deaconess Medical Center in 2001–2012 (15). This database collects some information, such as demographic characteristics, vital signs, laboratory tests, and imaging examinations (https://mimic.mit.edu/).

**Figure 1:**
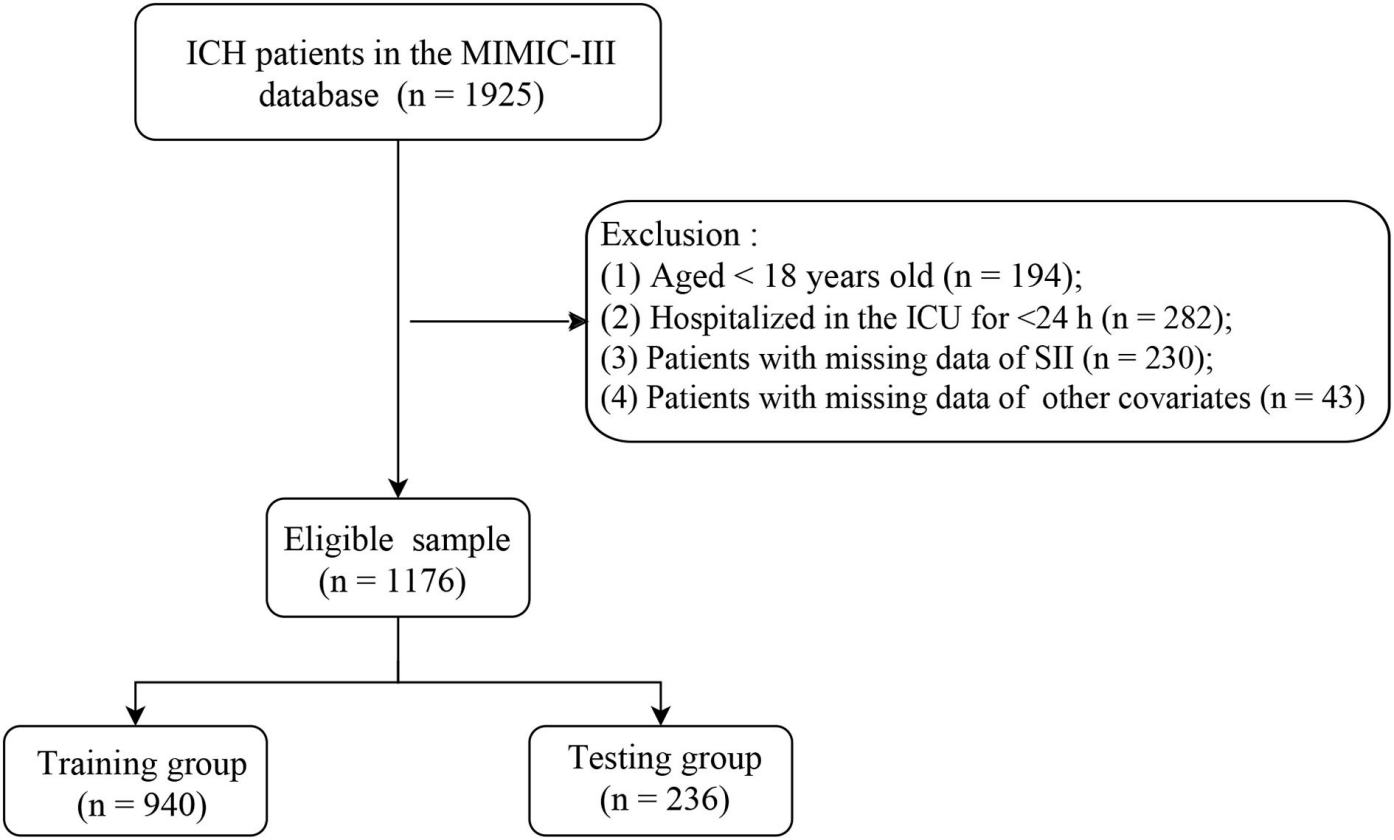
A flow diagram of the study.

Included criteria are as follows: The patients diagnosed with ICH according to International Classification of Diseases 9th revision (ICD-9) codes = 431, 432, 9487, and 77210–77214 from the MIMIC-III database. Excluded criteria are as follows: The patients who (1) aged <18 years; (2) were hospitalized in the ICU for <24 h; (3) had missing data of SII; and (4) had missing data of other covariates. According to these inclusion and exclusion criteria, a total of 1,176 eligible patients with ICH were ultimately included in this retrospective cohort study (Figure 1). Since this study used data from an anonymous public database that was approved by the Institutional Review Boards of Beth Israel Deaconess Medical Center (Boston, MA) and the Massachusetts Institute of Technology (Cambridge, MA), it was not necessary to obtain the approval of an ethics committee from the People's Hospital of Ningxia Hui Autonomous Region.

### Screening for potential predictors

In the present study, we extracted data about included patients from the MIMIC-III database (16, 17): age, gender, ethnicity, temperature (°C), heart rate (times/min), systolic blood pressure (SBP, mmHg), diastolic blood pressure (DBP, mmHg), mean artery pressure (MAP, mmHg), oxygen saturation (SpO2, %); the sequential organ failure assessment (SOFA) total score, the simplified acute physiology score II (SAPS II), the Glasgow Coma Scale (GCS), vasopressor, mechanical ventilation; comorbidities: chronic obstructive pulmonary disease (COPD), lung cancer, atrial fibrillation, liver cirrhosis, congestive heart failure (CHF), heart disease, diabetes, respiratory failure, hyperlipidemia, renal failure, malignant cancer, and hypertension; laboratory parameters: white blood cell count (WBC, K/μl), red blood cell (RBC, m/μl), sodium (mEq/L), potassium (mEq/L), calcium (mg/dl), magnesium (mg/dl), phosphate (mg/dl), platelet (K/μl), international normalized ratio (INR), mean corpuscular volume (MCV, fl), glucose (mg/dl), creatinine (Scr, mg/dl), blood urea nitrogen (BUN, mg/dl), bicarbonate (mEq/L), neutrophil (%), lymphocyte (%), hematocrit (%), hemoglobin (g/dl), mean corpuscular hemoglobin concentration (MCHC, %), red blood cell distribution width (RDW, %), and SII.

### Outcome and follow-up

The primary endpoint of this study was regarded as in-hospital death, and the starting point of follow-up was defined as the date of the patient's admission with a median follow-up time of 23.48 (14.83, 30.25) days. The SII index was calculated by the following formula: platelet × neutrophil/lymphocyte (13).

### Construction and validation of prediction nomogram

All included patients with ICH were randomly assigned to the training group for the construction of the nomogram and the testing group for the validation of the nomogram based on a ratio of 8:2. In the training group, predictors were screened by the least absolute shrinkage and selection operator (LASSO) regression analysis. These predictors were used to establish a prediction model. The receiver operator characteristic (ROC) curve was used to assess the predicting performance of the constructed nomogram.

### Statistical analysis

All data of patients were analyzed by descriptive statistics. The measurement data conducted the normal distribution and the *F*-test. If measurement data conform to the normal distribution and the *F*-test, mean ± standard deviation (Mean ± SD) was used to describe the data, and the comparison between the two groups was performed by Student's *t*-test. If measurement data do not conform to the normal distribution, we used the median and quartile spacing [M (Q1, Q3)] to present the data, and the comparison between the two groups was performed by the Mann–Whitney *U*-test. In addition, the categorical data were depicted by the number of cases and the composition ratio n (%), and the difference of groups was compared by the chi-square test. The statistical analyses were performed by using SAS 9.4 (SAS Institute Inc., Cary, NC, USA), R 4.2.0, and Python 3.7.4 software.

In the training group, the LASSO regression analysis (18), as a method of shrinkage and variable selection for linear regression models, was adopted to identify predictors, and the optimal penalty parameter was determined by the 10-fold cross-validation. A multivariate Cox regression analysis was used to investigate the association between SII and in-hospital death of patients with ICH in the ICU and developed a model for predicting the in-hospital death risk for ICU patients with ICH by combining the predictors selected in the LASSO regression analysis. Additionally, we compared the predicting performance between the developed nomogram and the traditional scoring systems (SOFA and SAPSII). The hazard ratio (*HR*) and 95% confidence interval (CI) were calculated, and *p* < 0.05 was considered to be statistically significant. If the missing data of a variable were <5%, the samples with these missing data were deleted in our study, and the sensitivity analysis was performed in the population before and after deletion (Supplementary Table 1).

## Results

### Characteristics of patients with ICH

A total of 1,176 patients with ICH were included in this study. These eligible patients with ICH were randomly divided into the training group (*n* = 940) and the testing group (*n* = 236). In the training group, 232 patients with ICH died and 708 survived, and the baseline characteristics of patients are listed in Table 1. The patients with ICH in the death group, with a mean age of 69.53 years, were older than the survival group of 66.72 years. We found that the patients with ICH in the survival group were more likely to suffer from respiratory failure, renal failure, and mechanical ventilation than those in the death group. In addition, the patients with ICH in the death group had a higher SII index compared with the survival group.

**Table 1:** The characteristics of included patients in the training group.

**Variables**	**Total (*n* = 940)**	**Survival group (*n* = 708)**	**Death group (*n* = 232)**	**Statistics**	***P***
Age, years, Mean ± SD	67.41 ± 15.28	66.72 ± 15.33	69.53 ± 14.97	*t* = −2.44	0.015
Gender, *n* (%)				χ^2^ = 0.475	0.491
Male	553 (58.83)	421 (59.46)	132 (56.90)		
Female	387 (41.17)	287 (40.54)	100 (43.10)		
Ethnicity, *n* (%)				χ^2^ = 0.693	0.707
White	696 (74.04)	527 (74.44)	169 (72.84)		
Black	70 (7.45)	54 (7.63)	16 (6.90)		
Other^#^	174 (18.51)	127 (17.94)	47 (20.26)		
Insurance, *n* (%)				χ^2^ = 8.127	0.017
Medicare	541 (57.55)	391 (55.23)	150 (64.66)		
Other*	125 (13.30)	94 (13.28)	31 (13.36)		
Private	274 (29.15)	223 (31.50)	51 (21.98)		
LOS, days, M (Q1, Q3)	3.15 (1.90, 6.99)	3.07 (1.88, 7.34)	3.44 (2.05, 6.14)	Z = 0.374	0.708
Temperature, °C, Mean ± SD	36.71 ± 1.51	36.69 ± 1.60	36.77 ± 1.22	*t* = −0.73	0.463
Heart rate, times/min, Mean ± SD	81.45 ± 17.33	80.22 ± 16.14	85.20 ± 20.11	*t* = −3.43	<0.001
SBP, mmHg, Mean ± SD	141.85 ± 25.60	142.53 ± 24.51	139.79 ± 28.64	*t* = 1.31	0.191
DBP, mmHg, Mean ± SD	71.75 ± 17.52	72.37 ± 17.02	69.87 ± 18.90	*t* = 1.79	0.074
MAP, mmHg, Mean ± SD	91.65 ± 17.97	92.49 ± 17.39	89.06 ± 19.46	*t* = 2.39	0.017
SPO2, %, Mean ± SD	97.98 ± 3.83	97.79 ± 4.20	98.53 ± 2.26	*t* = −3.42	<0.001
WBC, K/uL, M (Q_1_, Q_3_)	10.10 (7.60, 13.25)	9.80 (7.55, 12.60)	11.60 (8.40, 15.50)	Z = 4.312	<0.001
RBC, m/uL, Mean ± SD	4.23 ± 0.74	4.28 ± 0.69	4.07 ± 0.86	*t* = 3.38	<0.001
Sodium, mEq/L, Mean ± SD	138.61 ± 4.62	138.61 ± 4.34	138.58 ± 5.41	*t* = 0.09	0.925
Potassium, mEq/L, Mean ± SD	4.13 ± 0.82	4.12 ± 0.76	4.15 ± 0.97	*t* = −0.41	0.685
Phosphate, mg/dL, M (Q_1_, Q_3_)	3.20 (2.70, 3.80)	3.20 (2.70, 3.70)	3.10 (2.70, 3.90)	Z = −0.623	0.533
Calcium, mg/dL, Mean ± SD	8.86 ± 0.81	8.88 ± 0.76	8.78 ± 0.93	*t* = 1.42	0.156
Platelet, K/uL, M (Q_1_, Q_3_)	230.00 (176.00, 287.50)	232.50 (178.00, 287.00)	223.50 (158.00, 298.00)	Z = −1.199	0.231
INR, ratio, M (Q_1_, Q_3_)	1.10 (1.10, 1.40)	1.10 (1.10, 1.30)	1.20 (1.10, 1.60)	Z = 2.801	0.005
MCV, fL, Mean ± SD	89.45 ± 6.48	89.11 ± 6.10	90.48 ± 7.42	*t* = −2.55	0.011
Magnesium, mg/dL, Mean ± SD	1.94 ± 0.32	1.95 ± 0.31	1.91 ± 0.36	*t* = 1.18	0.238
Glucose, mg/dL, M (Q_1_, Q_3_)	134.00 (109.00, 169.00)	129.00 (108.00, 163.00)	150.00 (118.00, 198.00)	Z = 5.190	<0.001
Creatinine, mg/dL, M (Q_1_, Q_3_)	0.90 (0.80, 1.20)	0.90 (0.80, 1.10)	1.00 (0.80, 1.30)	Z = 1.574	0.115
BUN, mg/dL, M (Q_1_, Q_3_)	18.00 (13.00, 24.00)	17.00 (13.00, 23.00)	20.00 (15.00, 27.50)	Z = 4.050	<0.001
Bicarbonate, mEq/L, Mean ± SD	24.69 ± 3.76	24.86 ± 3.60	24.16 ± 4.15	*t* = 2.30	0.022
Neutrophil, %, Mean ± SD	78.09 ± 13.77	77.66 ± 13.08	79.40 ± 15.66	*t* = −1.52	0.129
Lymphocytes, %, M (Q_1_, Q_3_)	12.20 (7.80, 19.20)	13.25 (8.65, 20.15)	10.00 (5.95, 16.20)	Z = −5.076	<0.001
Hematocrit, %, Mean ± SD	37.61 ± 6.07	37.96 ± 5.74	36.55 ± 6.88	*t* = 2.82	0.005
Hemoglobin, g/dL, Mean ± SD	12.83 ± 2.18	12.97 ± 2.06	12.39 ± 2.46	*t* = 3.25	0.001
MCHC, %, Mean ± SD	34.08 ± 1.41	34.15 ± 1.42	33.86 ± 1.38	*t* = 2.78	0.006
RDW, %, Mean ± SD	14.39 ± 1.87	14.22 ± 1.75	14.92 ± 2.11	*t* = −4.53	<0.001
SII, M (Q_1_, Q_3_)	1520.30 (788.22, 2607.95)	1409.97 (777.82, 2401.21)	1869.86 (848.88, 3453.13)	Z = 3.396	<0.001
SAPS II, M (Q1, Q3)	34.00 (27.00, 44.00)	32.50 (26.00, 40.00)	43.00 (35.00, 54.00)	Z = 11.167	<0.001
SOFA, M (Q1, Q3)	4.00 (2.00, 6.00)	3.00 (2.00, 5.50)	6.00 (5.00, 8.00)	Z = 12.533	<0.001
GCS, M (Q1, Q3)	14.00 (11.00, 15.00)	14.00 (12.00, 15.00)	15.00 (7.00, 15.00)	Z = −0.937	0.349
Vasopressor, yes, n (%)	34 (3.62)	5 (0.71)	29 (12.50)	χ^2^ = 69.719	<0.001
Mechanical ventilation, yes, n (%)	535 (56.91)	337 (47.60)	198 (85.34)	χ^2^ = 101.527	<0.001
COPD, yes, n (%)	69 (7.34)	53 (7.49)	16 (6.90)	χ^2^ = 0.089	0.765
Lung cancer, yes, n (%)	25 (2.66)	22 (3.11)	3 (1.29)	χ^2^ = 2.222	0.136
Atrial fibrillation, yes, n (%)	235 (25.00)	171 (24.15)	64 (27.59)	χ^2^ = 1.099	0.295
Liver cirrhosis, yes, n (%)	25 (2.66)	10 (1.41)	15 (6.47)	χ^2^ = 17.235	<0.001
CHF, yes, n (%)	154 (16.38)	107 (15.11)	47 (20.26)	χ^2^ = 3.377	0.066
Heart disease, yes, n (%)	39 (4.15)	26 (3.67)	13 (5.60)	χ^2^ = 1.639	0.201
Diabetes, yes, n (%)	182 (19.36)	139 (19.63)	43 (18.53)	χ^2^ = 0.135	0.713
Respiratory failure, yes, n (%)	222 (23.62)	152 (21.47)	70 (30.17)	χ^2^ = 7.338	0.007
Hyperlipidemia, yes, n (%)	273 (29.04)	216 (30.51)	57 (24.57)	χ^2^ = 2.991	0.084
Renal failure, yes, n (%)	138 (14.68)	92 (12.99)	46 (19.83)	χ^2^ = 6.514	0.011
Malignant cancer, yes, n (%)	189 (20.11)	156 (22.03)	33 (14.22)	χ^2^ = 6.635	0.010
Hypertension, yes, n (%)	653 (69.47)	492 (69.49)	161 (69.40)	χ^2^ = 0.001	0.978
Time, days, M (Q_1_, Q_3_)	23.54 (14.23, 30.38)	25.15 (18.14, 31.92)	14.94 (8.80, 23.38)	Z = −10.024	<0.001

Other^#^: Asian, Hispanic, and so on; Other^*^: Government, Medicaid, and self-pay; LOS, length of stay; SBP, systolic blood pressure; DPB, diastolic blood pressure; MAP, mean artery pressure; SpO2, oxygen saturation; SAPS II, simplified acute physiology score II; SOFA, sequential organ failure assessment; GCS, Glasgow Coma Scale; COPD, chronic obstructive pulmonary disease; CHF, congestive heart failure; WBC, white blood cell count; RBC, red blood cell; SII, systemic immune-inflammation; INR, international normalized ratio; MCV, mean corpuscular volume; Scr, creatinine; BUN, blood urea nitrogen; MCHC, mean corpuscular hemoglobin concentration; RDW, red blood cell distribution width; χ^2^: Chi-square test; Z: Mann–Whitney U rank sum test.

### SII was associated with the risk of in-hospital death in ICU patients with ICH

**Figure 2:**
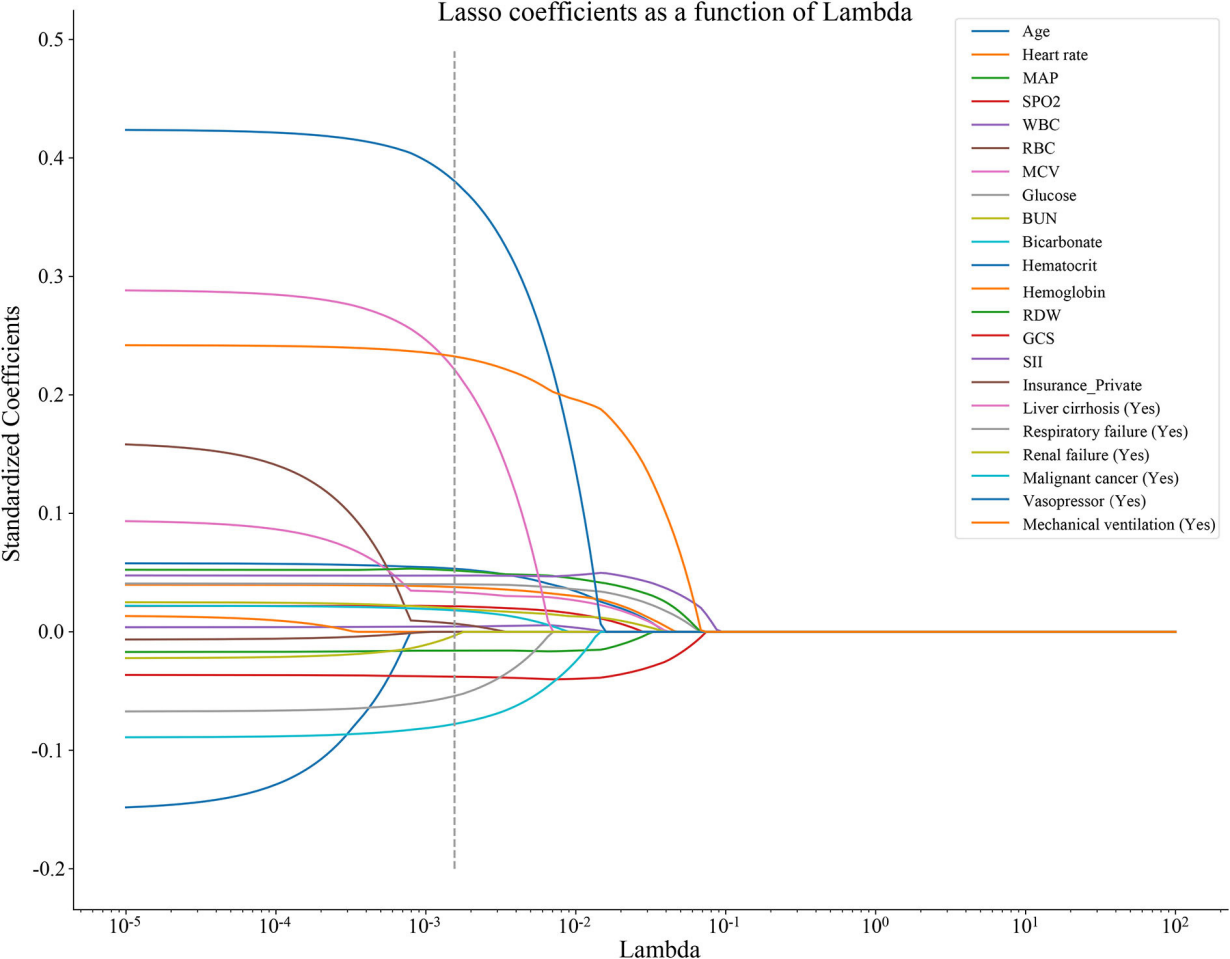
The predictors were selected in the LASSO regression analysis by the 10-fold cross-validation, and the optimal penalty parameter was 0.00155. LASSO, least absolute shrinkage and selection operator; MAP, mean artery pressure; SpO2, oxygen saturation; WBC, white blood cell count; RBC, red blood cell; MCV, mean corpuscular volume; BUN, blood urea nitrogen; RDW, red blood cell distribution width; GCS, Glasgow Coma Scale; and SII, systemic immune-inflammation.

As shown in Figure 2, 17 predictors were identified through the LASSO regression analysis. These predictors included WBC, glucose, BUN, SII, GCS, age, heart rate, MAP, RBC, bicarbonate, RDW, liver cirrhosis, respiratory failure, renal failure, malignant cancer, vasopressor, and mechanical ventilation. The result of the multivariate Cox regression analysis showed (Table 2) that SII was associated with the risk of in-hospital death among patients with ICH in the ICU. Notably, SII might be an independent risk factor of in-hospital death for patients with ICH in the ICU, with an *HR* of 1.12 (95% *CI*: 1.02–1.22).

**Table 2:** The HR of the selected predictor in multivariate Cox regression analysis.

**Variables**	**HR (95%CI)**	***P***
Age	1.54 (1.32, 1.81)	<0.001
MAP	0.95 (0.83, 1.09)	0.440
Heart rate	1.16 (1.03, 1.32)	0.019
GCS	0.85 (0.76, 0.95)	0.004
WBC	1.01 (0.94, 1.09)	0.716
Glucose	1.18 (1.07, 1.32)	0.001
BUN	0.99 (0.86, 1.13)	0.840
SII	1.12 (1.02, 1.22)	0.013
RBC	0.96 (0.83, 1.11)	0.569
Bicarbonate	1.09 (0.96, 1.25)	0.185
RDW	1.22 (1.08, 1.38)	0.001
Vasopressor, yes	3.60 (2.22, 5.83)	<0.001
Mechanical ventilation, yes	3.99 (2.69, 5.91)	<0.001
Liver cirrhosis, yes	2.36 (1.31, 4.24)	0.004
Respiratory failure, yes	0.56 (0.40, 0.76)	<0.001
Renal failure, yes	0.84 (0.55, 1.27)	0.409
Malignant cancer, yes	0.73 (0.50, 1.07)	0.104

HR, hazard ratio; CI, confidence interval; MAP, mean artery pressure; GCS, Glasgow Coma Scale; WBC, white blood cell count; BUN, blood urea nitrogen; SII, systemic immune-inflammation; RBC, red blood cell; RDW, red blood cell distribution width.

### Development and validation of prediction nomogram

**Figure 3:**
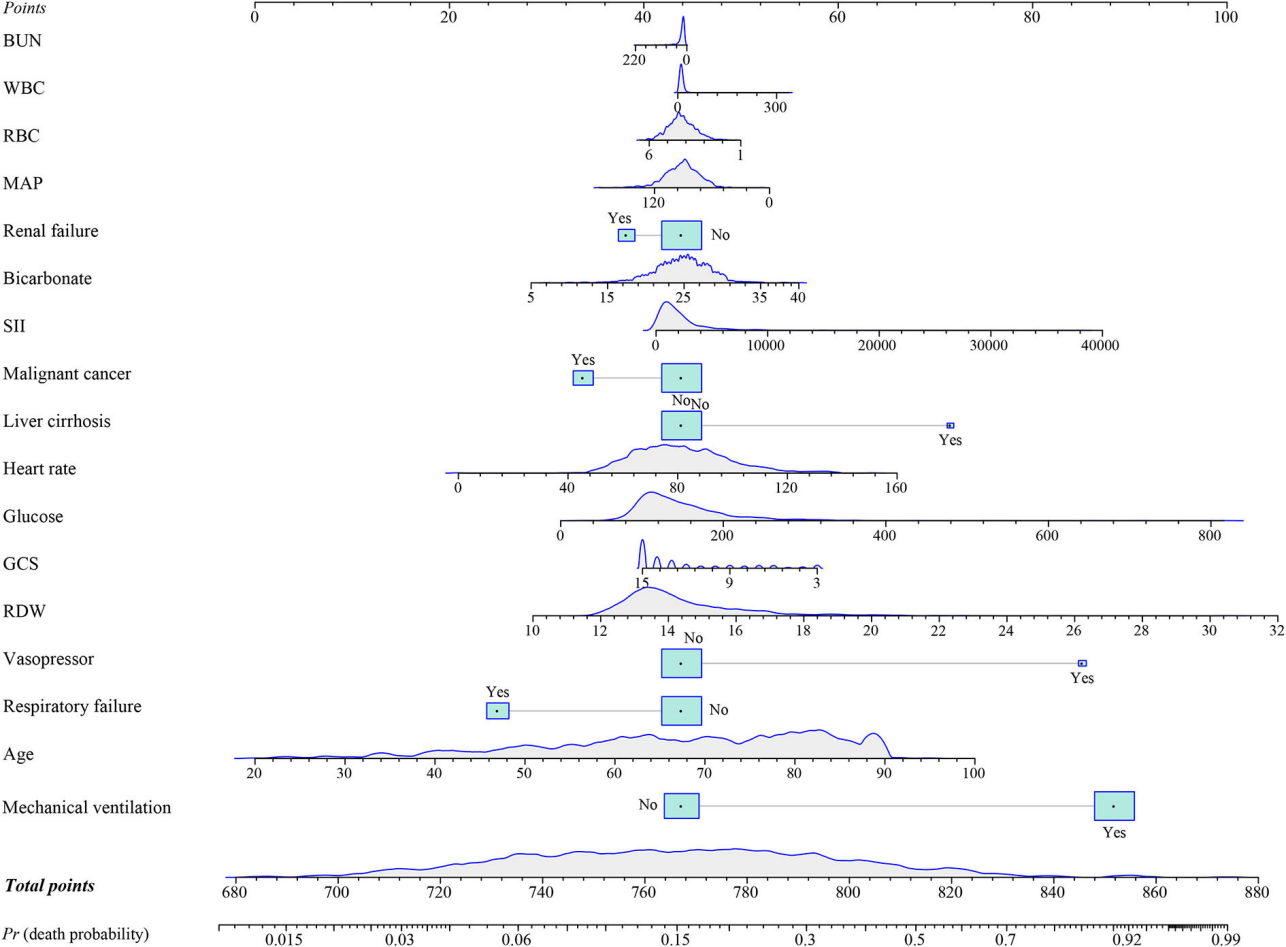
A nomogram to predict the risk of in-hospital death for patients with intracerebral hemorrhage (ICH). BUN, blood urea nitrogen; WBC, white blood cell count; RBC, red blood cell; MAP, mean artery pressure; SII, systemic immune-inflammation; GCS, Glasgow Coma Scale; and RDW, red blood cell distribution width.

**Figure 4:**
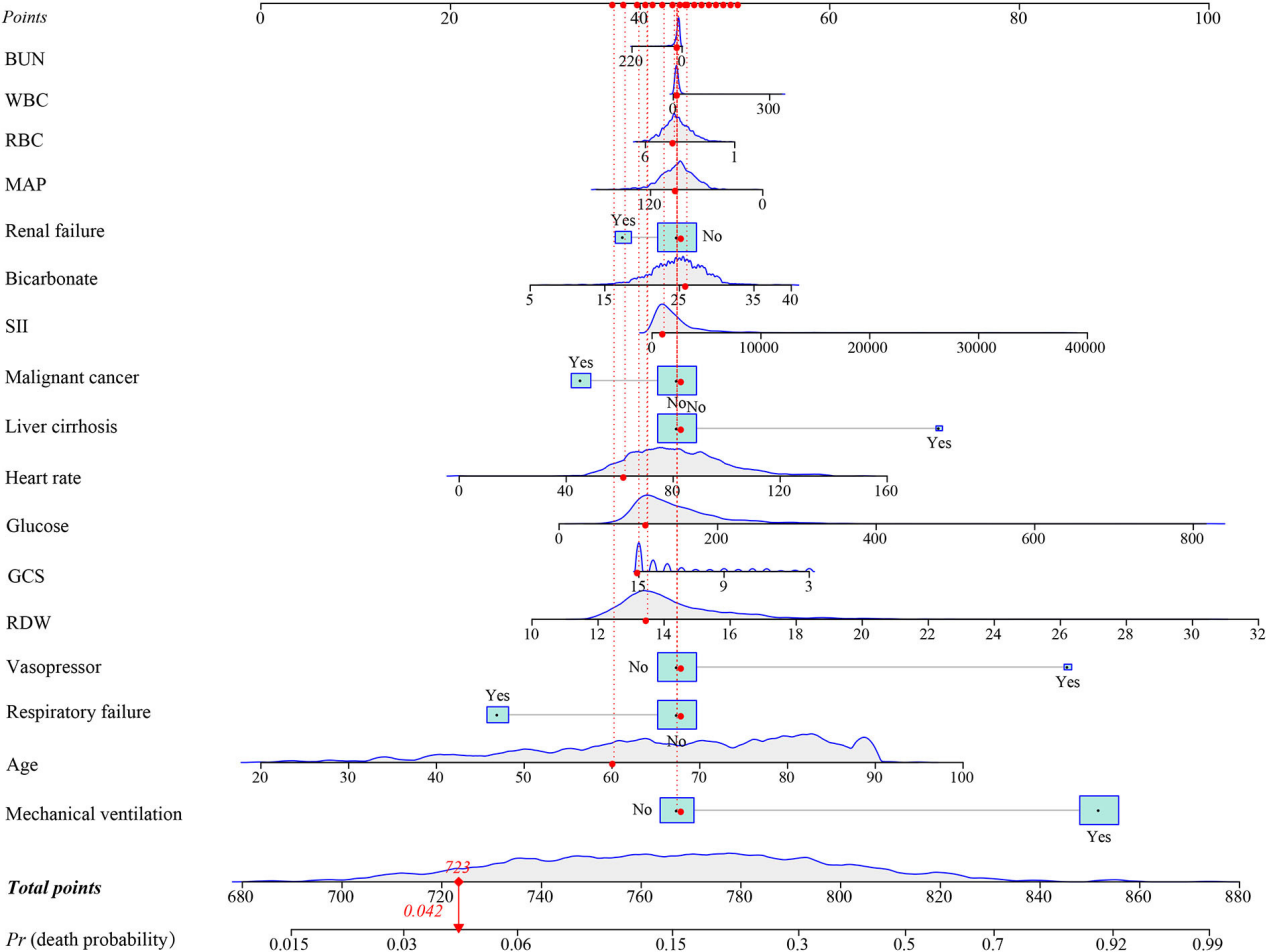
An example for the application of the nomogram. BUN, blood urea nitrogen; WBC, white blood cell count; RBC, red blood cell; MAP, mean artery pressure; SII, systemic immune-inflammation; GCS, Glasgow Coma Scale; and RDW, red blood cell distribution width.

In the training group, a prediction model integrating WBC, glucose, BUN, SII, GCS, age, heart rate, MAP, RBC, bicarbonate, RDW, liver cirrhosis, respiratory failure, renal failure, malignant cancer, vasopressors, and mechanical ventilation was established. Additionally, for visualizing the prediction model, we plotted a nomogram to predict the probability of in-hospital death for patients with ICH (Figure 3). For example, as shown in Figure 4, the patient was 60 years old with a heart rate of 62 times/min. The MAP was 92 mmHg and the WBC was 15.9 K/μl. The RBC was 4.38 m/μl and the glucose was 111 mg/dl. The BUN was 16 mg/dl and the bicarbonate was 26 mEq/L. The RDW was 13.5% and the GCS was 15. The patient had no complications with liver cirrhosis, respiratory failure, renal failure, or malignant cancer. In addition, the patient did not use vasopressors and mechanical ventilation. According to the nomogram, the total point was 723 and the corresponding predicted probability was 0.042, which indicated a lower risk of death and was in line with the actual outcome of the patient. An online prediction nomogram is available: https://os-model.shinyapps.io/DynNomapp/.

**Figure 5:**
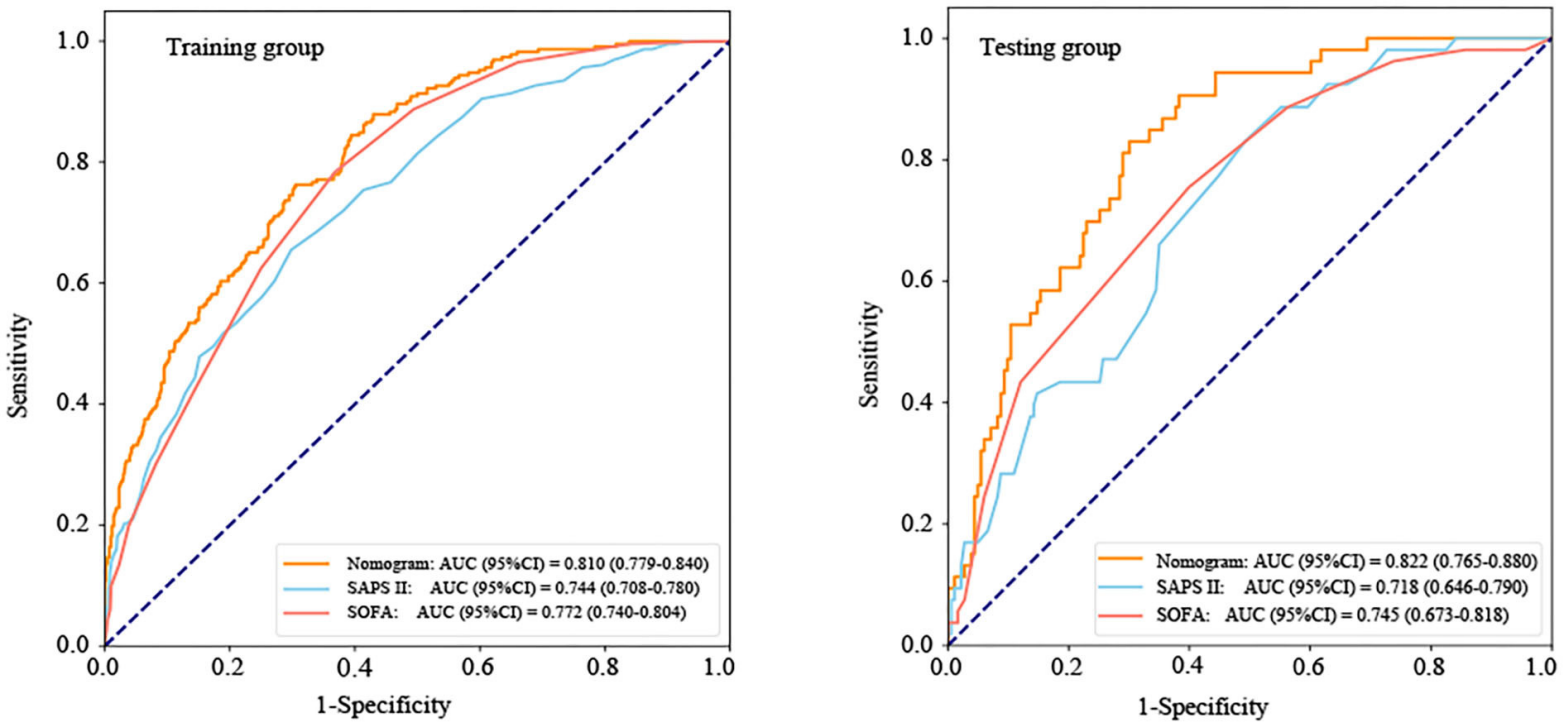
The receiver operator characteristic (ROC) curves of the nomogram and other scoring system. AUC, area under the curve; CI, confidence interval; SOFA, sequential organ failure assessment; and SAPS II, simplified acute physiology score II.

According to the ROC analysis (Figure 5 and Table 3), the area under the curve (AUC) of the nomogram (training group: AUC = 0.810, 95% *CI*: 0.779–0.840; testing group: AUC = 0.822, 95% *CI*: 0.765–0.880) was significantly higher than the SOFA (training group: AUC = 0.772, 95% *CI*: 0.740–0.804; testing group: AUC = 0.745, 95% *CI*: 0.673–0.818) and SAPS II (training group: AUC = 0.744, 95% *CI*: 0.708–0.780; testing group: AUC = 0.718, 95% *CI*: 0.646–0.790) scores. Furthermore, Table 3 also displays that the nomogram has good accuracy, specificity, sensitivity, positive predictive value (PPV), and negative predictive value (NPV) in recognizing whether the ICU patients with ICH survived or were in-hospital death, with 0.712 (95% *CI*: 0.683–0.741), 0.695 (95% *CI*: 0.661–0.729), 0.763 (95% *CI*: 0.708–0.818), 0.450 (95% *CI*: 0.401–0.500), and 0.899 (95% *CI*: 0.874–0.925) in the training group and 0.695 (95% *CI*: 0.636–0.754), 0.650 (95% *CI*: 0.581–0.719), 0.849 (95% *CI*: 0.753–0.945), 0.413 (95% *CI*: 0.320–0.505), and 0.937 (95% *CI*: 0.895–0.979) in the testing group, respectively.

**Table 3:** Predictive value of the nomogram.

		**Cut off**	**AUC (95%CI)**	**Accuracy (95%CI)**	**Specificity (95%CI)**	**Sensitivity (95%CI)**	**PPV (95%CI)**	**NPV (95%CI)**
**Nomogram**	Training group	0.701	0.810 (0.779–0.840)	0.712 (0.683–0.741)	0.695 (0.661–0.729)	0.763 (0.708-0.818)	0.450 (0.401-0.500)	0.899 (0.874-0.925)
	Testing group		0.822 (0.765–0.880)	0.695 (0.636–0.754)	0.650 (0.581–0.719)	0.849 (0.753–0.945)	0.413 (0.320–0.505)	0.937 (0.895–0.979)
**SOFA**	Training group	0.522	0.772 (0.740–0.804)	0.669 (0.639–0.699)	0.631 (0.596–0.667)	0.784 (0.732–0.837)	0.411 (0.365–0.457)	0.899 (0.873–0.926)
	Testing group		0.745 (0.673–0.818)	0.712 (0.654–0.770)	0.749 (0.686–0.811)	0.585 (0.452–0.718)	0.403 (0.293–0.512)	0.862 (0.808–0.915)
**SAPSII**	Training group	0.521	0.744 (0.708–0.780)	0.690 (0.661–0.720)	0.702 (0.668–0.736)	0.655 (0.594–0.716)	0.419 (0.368–0.469)	0.861 (0.833–0.890)
	Testing group		0.718 (0.646–0.790)	0.640 (0.579–0.701)	0.656 (0.587–0.725)	0.585 (0.452–0.718)	0.330 (0.235–0.425)	0.845 (0.786–0.905)

SAPS II, simplified acute physiology score II; SOFA, sequential organ failure assessment; AUC, area under the curve; PPV, positive predictive value; NPV, negative predictive value; CI, confidence interval.

## Discussion

In this retrospective cohort study, we found that SII was an independent risk factor of in-hospital death for patients with ICH in the ICU. A prediction model integrating WBC, glucose, BUN, SII, GCS, age, heart rate, MAP, RBC, bicarbonate, RDW, liver cirrhosis, respiratory failure, renal failure, malignant cancer, vasopressors, and mechanical ventilation was established. To our knowledge, this is the first study to explore the relationship between SII and the in-hospital death risk of patients with ICH in the ICU and develop and validate a nomogram associated with SII to predict the risk of in-hospital death among patients with ICH in the ICU. This nomogram showed a good performance to predict the in-hospital death risk for patients with ICH in the ICU in both the training and testing groups.

Inflammatory molecules, as key participants in the secondary injury process after ICH, were also associated with a poor prognosis of ICH (19). For example, an increased C-reactive protein (CRP) level has been found to be a risk factor for predicting an unfavorable prognosis of primary ICH (20). In the study of Miao et al., Interleukin 33 (IL-33) on admission may also be a prognostic indicator of ICH (21). Despite the fact that these inflammatory molecules showed a good performance value in predicting the prognosis of patients with ICH, their clinical application was limited due to the high cost of detection. SII, as an innovative inflammatory biomarker, has been proven to comprehensively reflect the inflammatory state in the body (22). The SII index is composed of neutrophils, lymphocytes, and platelets in which the three components are indicators of blood routine examination (23). In general, a blood routine is a routine examination for all critically ill hospitalized patients. Therefore, compared with other inflammatory indicators, the SII index is simpler, faster, and more inexpensive for clinical application (23).

In this study, the findings indicated that the SII index might be an independent risk factor for in-hospital death among patients with ICH in the ICU, and the higher the SII level, the higher the risk of in-hospital mortality may be. The mechanisms behind the association of SII and poor prognosis of ICH are still unclear so far, and the explanation might be as follows: an increase in SII levels implies a relative increase of platelet and neutrophil counts or a relative decrease of lymphocytes. Platelets are considered an important component of the hemostatic system (24). When patients suffer from ICH, the platelet count in the peripheral circulation increase, leading to a hypercoagulable state that might increase the risk of adverse outcomes (25, 26). In general, damage to vulnerable parts of the central nervous system may increase the sympathetic system or the hypothalamic-pituitary-adrenal axis, which may favor apoptosis of peripheral lymphocytes and lead to a decrease in the number of lymphocytes, thus contributing to a lower immune capacity and a higher risk of infection (27). Neutrophils can induce neurotoxicity through the release of pro-inflammatory cytokines, free radicals, and other toxic chemicals, which further lead to the destruction of the blood-brain barrier and exacerbate the brain damage caused by ICH (26–28).

Recently, a nomogram has been widely used as a prognostic tool to predict individual probabilities of clinical events by using individual variables (29). For the in-hospital death risk of patients with ICH in the ICU, our study is the first to provide a simple and effective nomogram related to SII. The clinician could calculate the score of each indicator and then add up each score to get the final total score, which could correspond to the probability of in-hospital death risk in the nomogram. We believed that the nomogram might help clinicians to quickly and easily understand the risk of death of patients and make timely individualized prevention. Not only that, the nomogram also might show good predicting performance in the in-hospital death risk of patients with ICH in the ICU by the ROC analysis, calibration curve, and DCA. In addition, we compared the predictive ability between the proposed nomogram and the two traditional predictive scoring systems (SOFA and SAPS II). The result also found that the nomogram showed a better predictive ability than the SOFA and SAPS II.

However, the present study had several limitations. First, as a single-center and retrospective study, the sample size was relatively small. Although the nomogram might have a good performance for predicting the death risk of in-hospital patients with ICH in the ICU through internal verification, this nomogram was constructed based on the U.S. population who were hospitalized in the ICU for >24 h, and these patients may be admitted to the ICU as a result of a variety of other factors, such as self-selecting for patients with a worse prognosis. Thus, its generalizability to other populations was still unclear. More prospective and multi-center studies are needed to confirm this result in the future. Second, because all data of this study were derived from the MIMIC-III database, and we could not obtain the imaging features of ICH (such as hematoma volume, hematoma location, intraventricular hemorrhage, intraventricular extension, midline shift, and brainstem extension), and some inflammatory markers (such as C-reactive protein, interleukin-6, and tumor necrosis factor-α), but, the established nomogram contained some indicators that are easy to obtain, and the use of the model has lower professional requirements for medical staff, which makes it more convenient for application in clinical practice. Third, we excluded some patients with missing data, this was also a significant limitation of this study. Fourth, the ICH score (30), as a simple and reliable grading scale for ICH, was not recorded in the MIMIC-III database. Therefore, we did not compare the performance of the nomogram and the ICH score. Last, external validation should be still required in the future.

## Conclusion

In summary, SII was associated with an increased in-hospital death risk for patients with ICH in the ICU. Importantly, we developed and validated a nomogram for the risk of in-hospital death for patients with ICH in the ICU, including WBC, glucose, BUN, SII, GCS, age, heart rate, MAP, RBC, bicarbonate, RDW, liver cirrhosis, respiratory failure, renal failure, malignant cancer, vasopressors, and mechanical ventilation. Additionally, the nomogram showed good performance in both the training and testing groups. We believed that the developed nomogram will be a simple and convenient tool to help clinicians accurately identify ICH patients with a high in-hospital death risk.

## Data availability statement

The data analyzed in this study was obtained from the Medical Information Mart for Intensive Care III (MIMIC-III) database, the following licenses/restrictions apply: To access the files, users must be a credentialed user, finish the required training and sign the data use agreement for the project. Requests to access these datasets should be directed to PhysioNet, https://physionet.org/, https://doi.org/10.13026/C2XW26.

## Ethics statement

Ethical review and approval was not required for the study on human participants in accordance with the local legislation and institutional requirements. Written informed consent from the patients/participants or patients/participants' legal guardian/next of kin was not required to participate in this study in accordance with the national legislation and the institutional requirements.

## Author contributions

LH and XL designed the study. LH wrote the manuscript. JY, JD, HZ, and FY collected, analyzed, and interpreted the data. XL critically reviewed, edited, and approved the manuscript. All authors read and approved the final manuscript.

## Conflict of interest

The authors declare that the research was conducted in the absence of any commercial or financial relationships that could be construed as a potential conflict of interest.

## Publisher's note

All claims expressed in this article are solely those of the authors and do not necessarily represent those of their affiliated organizations, or those of the publisher, the editors and the reviewers. Any product that may be evaluated in this article, or claim that may be made by its manufacturer, is not guaranteed or endorsed by the publisher.
