# Supplementary material for: Development of nomogram to predict in-hospital death for patients with intracerebral hemorrhage: A retrospective cohort study
Source: Front Neurol. 2022 Nov 24;13:968623. doi: 10.3389/fneur.2022.968623 (PMC9729245; doi:10.3389/fneur.2022.968623)
Supplement: Supplementary file 1 [file Table_1.docx]

**Supplemental Table 1 Sensitivity analysis of the population before and after deletion**

| **Variables** | **Total (n=2395)** | **Before deletion (n=1219)** | **After deletion (n=1176)** | **Statistics** | ***P*** |
| --- | --- | --- | --- | --- | --- |
| Age, years, Mean ± SD | 67.97 ± 15.13 | 68.00 ± 15.12 | 67.94 ± 15.14 | t=0.09 | 0.924 |
| Gender, n (%) |  |  |  | χ^2^=0.010 | 0.919 |
| Male | 1362 (56.87) | 692 (56.77) | 670 (56.97) |  |  |
| Female | 1033 (43.13) | 527 (43.23) | 506 (43.03) |  |  |
| Ethnicity, n (%) |  |  |  | χ^2^=0.022 | 0.989 |
| White | 1748 (72.99) | 889 (72.93) | 859 (73.04) |  |  |
| Black | 173 (7.22) | 89 (7.30) | 84 (7.14) |  |  |
| Other^#^ | 474 (19.79) | 241 (19.77) | 233 (19.81) |  |  |
| Insurance, n (%) |  |  |  | χ^2^=0.083 | 0.959 |
| Medicare | 1410 (58.87) | 720 (59.06) | 690 (58.67) |  |  |
| Other* | 311 (12.99) | 156 (12.80) | 155 (13.18) |  |  |
| Private | 674 (28.14) | 343 (28.14) | 331 (28.15) |  |  |
| LOS, days, M (Q1, Q3) | 3.14 (1.88, 7.10) | 3.11 (1.88, 7.09) | 3.17 (1.89, 7.11) | Z=0.439 | 0.660 |
| Temperature, ℃, M (Q1, Q3) | 36.69 ± 1.42 | 36.70 ± 1.42 | 36.69 ± 1.43 | t=0.11 | 0.909 |
| Heart rate, times/min, M (Q1, Q3) | 81.38 ± 17.41 | 81.40 ± 17.40 | 81.35 ± 17.42 | t=0.06 | 0.949 |
| SBP, mmHg, M (Q1, Q3) | 142.13 ± 25.41 | 142.18 ± 25.51 | 142.08 ± 25.31 | t=0.10 | 0.920 |
| DBP, mmHg, M (Q1, Q3) | 71.37 ± 17.33 | 71.35 ± 17.35 | 71.39 ± 17.31 | t=-0.06 | 0.953 |
| MAP, mmHg, Mean ± SD | 91.43 ± 17.60 | 91.42 ± 17.62 | 91.44 ± 17.59 | t=-0.04 | 0.971 |
| SPO2, %, Mean ± SD | 97.83 ± 4.59 | 97.82 ± 4.74 | 97.84 ± 4.44 | t=-0.14 | 0.890 |
| WBC, K/uL, M (Q_1_, Q_3_) | 10.10 (7.70, 13.30) | 10.10 (7.70, 13.30) | 10.10 (7.70, 13.30) | Z=-0.117 | 0.907 |
| RBC, m/uL, Mean ± SD | 4.22 ± 0.73 | 4.22 ± 0.72 | 4.22 ± 0.73 | t=-0.15 | 0.880 |
| Sodium, mEq/L, Mean ± SD | 138.70 ± 4.53 | 138.69 ± 4.54 | 138.70 ± 4.53 | t=-0.09 | 0.931 |
| Potassium, mEq/L, Mean ± SD | 4.13 ± 0.80 | 4.13 ± 0.80 | 4.13 ± 0.81 | t=-0.05 | 0.956 |
| Phosphate, mg/dL, M (Q_1_, Q_3_) | 3.32 ± 1.10 | 3.32 ± 1.10 | 3.32 ± 1.09 | t=0.09 | 0.930 |
| Calcium, mg/dL, Mean ± SD | 8.85 ± 0.80 | 8.85 ± 0.80 | 8.85 ± 0.80 | t=-0.01 | 0.989 |
| Platelet, K/uL, M (Q_1_, Q_3_) | 230.00 (176.00, 289.00) | 230.00 (177.00, 291.00) | 230.50 (176.00, 288.00) | Z=-0.020 | 0.984 |
| INR, ratio, M (Q_1_, Q_3_) | 1.10 (1.10, 1.40) | 1.10 (1.10, 1.40) | 1.10 (1.10, 1.40) | Z=0.006 | 0.995 |
| MCV, fL, Mean ± SD | 89.41 ± 6.53 | 89.41 ± 6.52 | 89.40 ± 6.54 | t=0.03 | 0.976 |
| Magnesium, mg/dL, Mean ± SD | 1.90 (1.70, 2.10) | 1.90 (1.70, 2.10) | 1.90 (1.70, 2.10) | Z=0.001 | 0.999 |
| Glucose, mg/dL, M (Q_1_, Q_3_) | 135.00 (110.00, 171.00) | 135.00 (111.00, 171.00) | 135.00 (110.00, 170.00) | Z=-0.253 | 0.800 |
| Creatinine, mg/dL, M (Q_1_, Q_3_) | 0.90 (0.80, 1.20) | 0.90 (0.80, 1.20) | 0.90 (0.80, 1.20) | Z=0.014 | 0.989 |
| BUN, mg/dL, M (Q_1_, Q_3_) | 18.00 (14.00, 24.00) | 18.00 (14.00, 24.00) | 18.00 (13.50, 24.00) | Z=-0.177 | 0.859 |
| Bicarbonate, mEq/L, Mean ± SD | 24.74 ± 3.71 | 24.74 ± 3.73 | 24.74 ± 3.69 | t=-0.01 | 0.990 |
| Neutrophil, %, Mean ± SD | 78.09 ± 13.68 | 78.16 ± 13.63 | 78.02 ± 13.74 | t=0.24 | 0.811 |
| Lymphocytes, %, M (Q_1_, Q_3_) | 12.20 (7.80, 19.20) | 12.20 (7.70, 19.10) | 12.25 (7.80, 19.20) | Z=0.254 | 0.800 |
| Hematocrit, %, Mean ± SD | 37.52 ± 5.90 | 37.50 ± 5.88 | 37.53 ± 5.92 | t=-0.14 | 0.885 |
| Hemoglobin, g/dL, Mean ± SD | 12.78 ± 2.11 | 12.77 ± 2.11 | 12.79 ± 2.12 | t=-0.15 | 0.882 |
| MCHC, %, Mean ± SD | 34.05 ± 1.40 | 34.04 ± 1.39 | 34.05 ± 1.40 | t=-0.07 | 0.944 |
| RDW, %, Mean ± SD | 14.37 ± 1.83 | 14.37 ± 1.83 | 14.37 ± 1.82 | t=0.03 | 0.978 |
| SII, M (Q_1_, Q_3_) | 1510.69 (800.34, 2597.28) | 1519.23 (801.19, 2608.50) | 1504.42 (788.22, 2576.40) | Z=-0.252 | 0.801 |
| SAPS II, M (Q1, Q3) | 35.00 (28.00, 44.00) | 34.00 (27.00, 44.00) | 35.00 (28.00, 44.00) | Z=0.130 | 0.897 |
| SOFA, M (Q1, Q3) | 4.00 (2.00, 6.00) | 4.00 (2.00, 6.00) | 4.00 (2.00, 6.00) | Z=0.165 | 0.869 |
| GCS, M (Q1, Q3) | 12.27 ± 3.57 | 12.25 ± 3.59 | 12.28 ± 3.54 | t=-0.22 | 0.823 |
| Vasopressor, n (%) |  |  |  | χ^2^=0.003 | 0.954 |
| No | 2310 (96.45) | 1176 (96.47) | 1134 (96.43) |  |  |
| Yes | 85 (3.55) | 43 (3.53) | 42 (3.57) |  |  |
| Mechanical ventilation, n (%) |  |  |  | χ^2^=0.012 | 0.913 |
| No | 1023 (42.71) | 522 (42.82) | 501 (42.60) |  |  |
| Yes | 1372 (57.29) | 697 (57.18) | 675 (57.40) |  |  |
| COPD, n (%) |  |  |  | χ^2^=0.029 | 0.865 |
| No | 2218 (92.61) | 1130 (92.70) | 1088 (92.52) |  |  |
| Yes | 177 (7.39) | 89 (7.30) | 88 (7.48) |  |  |
| Lung cancer, n (%) |  |  |  | χ^2^=0.022 | 0.882 |
| No | 2329 (97.24) | 1186 (97.29) | 1143 (97.19) |  |  |
| Yes | 66 (2.76) | 33 (2.71) | 33 (2.81) |  |  |
| Atrial fibrillation, n (%) |  |  |  | χ^2^=0.010 | 0.921 |
| No | 1776 (74.15) | 905 (74.24) | 871 (74.06) |  |  |
| Yes | 619 (25.85) | 314 (25.76) | 305 (25.94) |  |  |
| Liver cirrhosis, n (%) |  |  |  | χ^2^=0.008 | 0.928 |
| No | 2325 (97.08) | 1183 (97.05) | 1142 (97.11) |  |  |
| Yes | 70 (2.92) | 36 (2.95) | 34 (2.89) |  |  |
| CHF, n (%) |  |  |  | χ^2^=0.005 | 0.943 |
| No | 1987 (82.96) | 1012 (83.02) | 975 (82.91) |  |  |
| Yes | 408 (17.04) | 207 (16.98) | 201 (17.09) |  |  |
| Heart disease, n (%) |  |  |  | χ^2^=0.002 | 0.967 |
| No | 2305 (96.24) | 1173 (96.23) | 1132 (96.26) |  |  |
| Yes | 90 (3.76) | 46 (3.77) | 44 (3.74) |  |  |
| Diabetes, n (%) |  |  |  | χ^2^=0.072 | 0.789 |
| No | 1909 (79.71) | 969 (79.49) | 940 (79.93) |  |  |
| Yes | 486 (20.29) | 250 (20.51) | 236 (20.07) |  |  |
| Respiratory failure, n (%) |  |  |  | χ^2^=0.013 | 0.910 |
| No | 1817 (75.87) | 926 (75.96) | 891 (75.77) |  |  |
| Yes | 578 (24.13) | 293 (24.04) | 285 (24.23) |  |  |
| Hyperlipidemia, n (%) |  |  |  | χ^2^=0.002 | 0.968 |
| No | 1720 (71.82) | 875 (71.78) | 845 (71.85) |  |  |
| Yes | 675 (28.18) | 344 (28.22) | 331 (28.15) |  |  |
| Renal failure, n (%) |  |  |  | χ^2^=0.007 | 0.934 |
| No | 2031 (84.80) | 1033 (84.74) | 998 (84.86) |  |  |
| Yes | 364 (15.20) | 186 (15.26) | 178 (15.14) |  |  |
| Malignant cancer, n (%) |  |  |  | χ^2^=0.018 | 0.894 |
| No | 1915 (79.96) | 976 (80.07) | 939 (79.85) |  |  |
| Yes | 480 (20.04) | 243 (19.93) | 237 (20.15) |  |  |
| Hypertension, n (%) |  |  |  | χ^2^=0.028 | 0.867 |
| No | 737 (30.77) | 377 (30.93) | 360 (30.61) |  |  |
| Yes | 1658 (69.23) | 842 (69.07) | 816 (69.39) |  |  |

Other^#^: Asian, Hispanic and so on; Other*: government, Medicaid and self-pay; LOS, length of stay; SBP, systolic blood pressure; DPB, diastolic blood pressure; MAP, mean artery pressure; SpO2, oxygen saturation; SAPS II, simplified acute physiology score II; SOFA, sequential organ failure assessment; GCS, Glasgow Coma Scale; COPD, chronic obstructive pulmonary disease; CHF, congestive heart failure; WBC, white blood cell count; RBC, red blood cell; SII, systemic immune-inflammation; INR, international normalized ratio; MCV, mean corpuscular volume; Scr, creatinine; BUN, blood urea nitrogen; MCHC, mean corpuscular hemoglobin concentration; RDW, red blood cell distribution width; χ^2^: Chi-square test; Z: Mann-Whitney U rank sum test.
